# Dapagliflozin and Sirtuin-1 interaction and mechanism for ameliorating atrial fibrillation in a streptozotocin-induced rodent diabetic model

**DOI:** 10.17305/bb.2024.11361

**Published:** 2024-11-28

**Authors:** Wei-Chieh Lee, Yu-Wen Lin, Jhih-Yuan Shih, Zhih-Cherng Chen, Nan-Chun Wu, Wei-Ting Chang, Ping-Yen Liu

**Affiliations:** 1 School of Medicine, College of Medicine, National Sun Yat-sen University, Kaohsiung, Taiwan; 2 Division of Cardiology, Department of Internal Medicine, Chi Mei Medical Center, Tainan, Taiwan; 3 Institute of Clinical Medicine, College of Medicine, National Cheng Kung University, Tainan, Taiwan; 4 Division of Cardiovascular Surgery, Department of Surgery, Chi Mei Medical Center, Tainan, Taiwan; 5 Department of Hospital and Health Care Administration, Chia Nan University of Pharmacy and Science, Tainan, Taiwan; 6 School of Medicine and Doctoral Program of Clinical and Experimental Medicine, College of Medicine and Center of Excellence for Metabolic Associated Fatty Liver Disease, National Sun Yat-sen University, Kaohsiung, Taiwan; 7 Division of Cardiology, Internal Medicine, National Cheng Kung University Hospital, College of Medicine, National Cheng Kung University, Tainan, Taiwan

**Keywords:** Sirtuin 1, SIRT1, dapagliflozin, atrial fibrillation, AF, apoptosis, sodium-glucose co-transporter-2 inhibitor, SGLT2i, calcium channel activity, reactive oxygen species, ROS

## Abstract

The incidence of atrial fibrillation (AF) increases with age and is particularly high in individuals with diabetes. Sodium-glucose cotransporter-2 inhibitors (SGLT2i), such as dapagliflozin, show promise in treating heart failure (HF) and reducing the risk of AF. Sirtuin 1 (SIRT1), a key enzyme in metabolic regulation, may be influenced by SGLT2i and play a role in the development of AF. This study investigates the relationship between dapagliflozin therapy and atrial tachyarrhythmia in diabetic cardiomyopathy, with a focus on the role of SIRT1. A streptozotocin (STZ)-induced diabetes mellitus (DM) rat model was used to assess AF across four groups: sham, STZ, STZ with dapagliflozin, and STZ with dapagliflozin + sirtinol (a SIRT1 inhibitor). Additionally, HL-1 cardiomyocytes were cultured under high glucose (HG) conditions and treated with dapagliflozin, with or without sirtinol. In the rat model, dapagliflozin improved atrial fibrosis and reduced AF inducibility and duration—effects that were partially reversed by sirtinol. These findings suggest that dapagliflozin may alleviate cardiac fibrosis and atrial arrhythmia by modulating SIRT1. In HL-1 cells under HG conditions, dapagliflozin reduced apoptosis, restored autophagy and mitophagy, and improved calcium channel activity. However, sirtinol negated these protective effects. Dapagliflozin helped normalize autophagy, mitophagy, and calcium handling, while sirtinol diminished its protective effects, highlighting the key role of SIRT1 in regulating calcium handling under HG conditions. Overall, SIRT1 plays a protective role in diabetic cardiomyopathy by reducing apoptosis, regulating autophagy and mitophagy, and modulating calcium channel activity. Dapagliflozin reduces AF duration and inducibility in the STZ model, likely through SIRT1 upregulation and calcium channel modulation.

## Introduction

Atrial fibrillation (AF) represents a significant clinical and public health challenge globally, primarily driven by the increasing prevalence associated with an aging population [1]. The current prevalence of AF in adults is estimated to range from 2%–4%, with projections indicating a 2.3-fold increase in the near future. This rise is attributed to the increased longevity of the general population and improved efforts to identify previously undiagnosed AF cases, along with advancements in medical treatment [2–4]. The lifetime risk of developing AF is influenced by various factors, including age, genetic predisposition, and coexisting conditions such as diabetes mellitus (DM) [5, 6]. Notably, AF affects approximately 25% of patients with DM and is associated with a poor prognosis [7, 8].

Currently, a novel class of oral hypoglycemic drugs, sodium-glucose co-transporter-2 inhibitors (SGLT2i), not only serve as antidiabetic treatments but have also shown significant benefits in patients with heart failure (HF) [9–12]. Recent randomized trials and meta-analyses have demonstrated that SGLT2i reduce the occurrence of atrial arrhythmias in patients with both HF and DM [13–15]. SGLT2i affect electrical remodeling by influencing Na^+^ and Ca2^+^ homeostasis, which plays a crucial role in reducing arrhythmia risk [16]. These changes in atrial electrical remodeling are associated with a decreased incidence of arrhythmogenesis. However, it is important to recognize that the risk of arrhythmia is often exacerbated by cardiac remodeling, hypertrophy, and dysfunction caused by fibrosis [17]. Atrial tachyarrhythmia, especially AF, is frequently observed in patients with atrial fibrosis, as shown by histopathological and magnetic resonance imaging findings [18]. Electrical and structural remodeling are the two primary forms of remodeling that contribute to the development of arrhythmias. However, no relevant studies have yet investigated the mechanisms underlying the reduction of AF through SGLT2i.

Oxidative stress occurs when there is an imbalance between the production and accumulation of reactive oxygen species (ROS) in cells and tissues and the ability of biological systems to detoxify and eliminate these harmful byproducts [19]. ROS are key contributors to the development and progression of AF, acting as mediators of AF-induced atrial remodeling [20]. Several inflammatory diseases, including hypertension, DM, HF, sleep apnea, and obesity, as well as lifestyle factors like smoking, can lead to increased ROS production, contributing to the imbalance that causes cellular and tissue damage [21]. Sirtuins, particularly Sirtuin 1 (SIRT1), play a crucial role in regulating oxidative stress by modulating the transcription of pro- and antioxidant genes and maintaining redox signaling pathways. SIRT1, an NAD+-dependent deacetylase, is involved in the regulation of oxidative stress, inflammation, and energy metabolism [22]. Elevated ROS levels directly and indirectly affect SIRT1 activity [23]. SIRT1 deficiency disrupts the regulation of intracellular calcium (Ca2^+^) and sodium (Na^+^) in cardiomyocytes, which can promote arrhythmogenesis [24]. SGLT2 inhibitors, such as dapagliflozin, have been shown to increase the levels of SIRT1, proliferator-activated receptor gamma coactivator 1α (PGC-1α), and fibroblast growth factor 21 (FGF21) in the heart [25]. Consequently, SGLT2 inhibitors may regulate SIRT1 activity, modulate autophagy flux, and reduce ROS levels, thereby influencing atrial fibrosis and AF through both direct and indirect mechanisms. This study aims to investigate the effects of dapagliflozin on AF control using a STZ-induced diabetic rat model. Specifically, it will focus on how dapagliflozin modulates calcium channel activity and its potential role in mitigating arrhythmogenesis associated with diabetes-induced atrial remodeling.

## Materials and methods

### Animal study design

Thirty-five male Sprague-Dawley rats (three months old, 200–250 g) were used in accordance with the guidelines of the Chi-Mei Medical Center and the Guide for the Care and Use of Laboratory Animals. The rats were randomly assigned to four groups: sham (*n* ═ 5), STZ (*n* ═ 10), STZ + DAPA (10 mg/kg/day orally for six weeks, *n* ═ 10), and STZ + DAPA + sirtinol (2 mg/kg/day orally for six weeks, *n* ═ 10). Diabetes was induced in fasting rats one week prior by administering an intravenous injection of STZ (35 mg/kg), and hyperglycemia was confirmed. The STZ-induced diabetic rat model followed methods from previous studies [26]. Blood glucose was measured 48–72 h after injection, with levels >250 mg/dL confirming diabetes. Throughout the 6-week study, blood glucose, body weight, cardiac function, heart rate (HR), and blood pressure were monitored bi-weekly.

### Echocardiographic measurements

The baseline echocardiography was performed one week prior to randomization and subsequently at two-week intervals using the GE Vivid S6 platform (GE Vingmed Ultrasound AS, Horten, Norway), equipped with a 10-MHz linear transducer. Rats were anesthetized with isoflurane to maintain an HR above 200 beats per minute, and images were acquired at a frame rate of 300–350 frames per second. Left ventricular (LV) M-mode and two-dimensional echocardiography in the parasternal short-axis view were used to evaluate parameters including the aortic (AO) annulus, left atrial (LA) diameter, fractional shortening (FS), interventricular septum thickness (IVSd), LV internal diameter (LVIDd), mitral valve (MV) velocities (E and A waves), MV E/A ratio, septal (SP) A’, E’, and SP E/E’. These measurements provided a comprehensive assessment of systolic and diastolic function. The protocol followed the methodology described in a previous study [27].

### Electrocardiographic (ECG) measurements

ECG recordings were obtained using a PowerLab converter (Millar Instruments, Houston, TX, USA) and analyzed with the LabChart system (AD Instruments, Dunedin, New Zealand). Rats were anesthetized with 3% isoflurane in oxygen to minimize the impact on HR. Lead II ECGs were recorded using needle electrodes placed on the four limbs [28]. The following measurements were taken: RR interval (time between successive R-wave peaks), PR interval (from the onset of the P wave to the peak of the R wave), P-wave duration (time until the return to baseline), QRS duration (from the start of the Q wave to the peak of the S wave), QT interval (from the start of the Q wave to the return of the T wave to baseline), and the corrected QT interval (QTc), which was calculated using Bazett’s formula: QTc-B ═ QT/(RR)ˆ(1/2).

### Pressure-volume loop hemodynamic analysis

Six weeks later, invasive hemodynamic assessments were performed using a Millar pressure catheter (SPR-838; Millar Instruments, Houston, TX, USA), following the methodology of a previous study [29]. Rats were anesthetized with intraperitoneal urethane (500 mg/kg). A 2.0 F pressure-volume catheter was inserted into the LV cavity via the right carotid artery, while the left jugular vein was cannulated for the infusion of hypertonic saline (10%) to assess conductance. The inferior vena cava was occluded with 3–0 silk sutures, and LV hemodynamic parameters were recorded using a PowerLab converter. Systolic function was evaluated by measuring end-systolic volume, pressure, maximal rate of pressure rise (+dP/dt), maximal rate of pressure fall (--dP/dt), arterial elastance, and the end-systolic pressure-volume relationship. Diastolic function was assessed by measuring end-diastolic volume, pressure, the relaxation time constant (tau), and the end-diastolic pressure-volume relationship.

### Atrial burst pacing for AF induction

Six weeks later, hearts were harvested and perfused through the aorta with Tyrode’s buffer (pH 7.4), containing NaCl, KCl, CaCl_2_, MgCl_2_, Na_2_HPO_ImEquation4_, HEPES, and glucose. The buffer was oxygenated with 95% O_2_ and 5% CO_2_, maintained at 37 ^∘^C, and perfused at a rate of 10–12 mL/min for 5 min. Silver bipolar electrodes were placed on the LA for burst pacing (3–10 Hz, 1 V, 15-ms pulse width), lasting 3–5 s. This protocol has been previously published [30], and the duration and inducibility of AF were subsequently recorded.

### Rat heart histological analysis

Histological analysis involved processing atrial tissues to evaluate myocyte morphology, fibrosis, and protein expression. Tissues were fixed in 4% paraformaldehyde, permeabilized with 0.1% Triton X-100, and stained with CytoPainter Phalloidin-iFluor 488 (Abcam). Nuclei were counterstained with Hoechst 33258 (Sigma). High-magnification images (400×) of four random fields, covering at least 100 cells, were captured using an Olympus DP80 microscope (Olympus Optical Co. Ltd., Tokyo, Japan) and analyzed with UTHSCSA Image Tool, Version 3.0. Fibrosis was assessed using Modified Masson’s Trichrome staining, which included fixation, staining with Weigert’s hematoxylin, Biebrich Scarlet/Acid Fuchsin, and differentiation in various solutions. Further analysis of HL-1 cells and atrial tissues examined fibrosis, apoptosis, and key proteins, including SIRT1 (Abcam, MA, USA), BCL-2/BAX (Arigo, Taiwan), Caspase 3 (Abcam, MA, USA), PTEN-induced kinase-1 (PINK-1) (Abclonal, MA, USA), Parkin (Abclonal, MA, USA), light chain 3-I (LC3-I) (Proteintech, IL, USA), Lysosome-associated membrane protein 2 (LAMP-2) (Proteintech, IL, USA), Mitofusin-2 (MFN2) (Proteintech, IL, USA), ryanodine receptor 2 (RyR2) (Badrilla, UK), Sarco/endoplasmic reticulum Ca^2+^-ATPase (SERCA2) (Santa Cruz, CA, USA), Connexin 43 (Abcam, MA, USA), and forkhead box O3 (FOXO3) (Abcam, MA, USA), to elucidate cellular and molecular changes under high-glucose conditions.

### TUNEL staining

Cardiac apoptosis was assessed using the TUNEL assay (BioVision, Milpitas, CA, USA). Formalin-fixed, paraffin-embedded heart tissue sections were deparaffinized, followed by antigen retrieval in citrate buffer (pH 6.0) for 10 min. The sections were then digested with proteinase K (20 mg/mL) at room temperature for 10 min. After digestion, the TUNEL reaction mixture was applied and incubated in the dark for 30 min. Sections were rinsed with phosphate-buffered saline (PBS) and counterstained with 4’,6-diamidino-2-phenylindole (DAPI; Vector Laboratories). Apoptotic cells were visualized using an Olympus BX51 fluorescence microscope. Ten randomly selected sections per heart were analyzed, with TUNEL-positive cells counted in three fields at 200× magnification. Results were expressed as the ratio of TUNEL-positive cells to total cells.

### Experimental design for cell culture studies

HL-1 murine atrial cardiomyocytes (American Type Culture Collection, CRL1446; Manassas, VA, USA) were cultured in Claycomb medium supplemented with 10% fetal bovine serum, 2-mM L-glutamine, 100 U/mL penicillin, 100 µg/mL streptomycin, and 0.1-mM norepinephrine. For high glucose (HG) treatment, cells were exposed to 30-mM glucose for 48 h, while control cells were maintained in 5.5-mM glucose. To assess the effects of dapagliflozin (DAPA) and sirtinol, HL-1 cells were pretreated with 20-µM DAPA for 2 h or 10-µM sirtinol for 1 h before HG exposure.

### Analysis of ROS

To assess ROS levels in HL-1 cells, a 2’,7’-dichlorofluorescin diacetate (DCFDA) assay was performed. Heart tissues were homogenized and incubated with 10-µM DCFDA for 30 min at 37 ^∘^C in the dark. The resulting fluorescent compound, dichlorofluorescein (DCF), which forms upon reaction with ROS, was detected using an Olympus BX51 fluorescence microscope. Tissues were then washed with PBS, mounted on slides, and imaged. Fluorescence intensity, reflecting ROS levels, was quantified using ImageJ software. Ten randomly selected fields per sample were analyzed, and ROS levels were expressed as fold changes relative to controls.

### Western blot analysis

Heart tissue proteins were separated by 8%–15% SDS-PAGE and transferred to polyvinylidene fluoride (PVDF) membranes (Merck Millipore). For Western blotting, membranes were blocked with 5% milk in Tris-buffered saline (pH 7.6) and incubated overnight at 4 ^∘^C with primary antibodies against proteins including SIRT1, FOXO3, cleaved Caspase 3, BAX, BCL2, LAMP-2, MFN2, PINK-1, Parkin, NIX, LC3-I, β-actin, RyR2, SERCA2, Connexin43, and GAPDH (1:1000 to 1:5000, from various suppliers). Membranes were then incubated with HRP-conjugated anti-rabbit/mouse IgG (1:5000) for 1 h at room temperature. Protein signals were detected using an enhanced chemiluminescence system (AVEGENE CHEMX 400), and band intensity was quantified using ImageJ software, with normalization to β-actin or GAPDH.

### Ethical statement

All animals were provided appropriate care in accordance with the Guide for the Care and Use of Laboratory Animals published by Taiwan’s National Institutes of Health. The study adhered to the principles outlined in the Declaration of Helsinki, and all animal procedures were reviewed and approved by the Institutional Animal Care and Use Committee (IACUC) of Chi Mei Medical Center (Approval Number: 111122106).

### Statistical analysis

Data are presented as the mean ± standard error of the mean (SEM). Group comparisons were conducted using one-way ANOVA, followed by Tukey’s post-hoc test for multiple comparisons. For experiments involving more than two groups, two-way ANOVA was used to assess interactions between factors, followed by post-hoc analysis to identify specific differences. Statistical significance was defined as *P* < 0.05. All graphs and statistical analyses were performed using GraphPad Prism (GraphPad Software Inc., La Jolla, CA, USA). Each experiment was conducted with at least three independent replicates to ensure result reliability. Quantitative data from western blotting and other assays were normalized to control values and expressed as fold changes relative to the control group.

## Results

### Echocardiographic, physiological, and ECG parameter changes over time in Sham, STZ, STZ+DAPA, and STZ+DAPA+Sirtinol treated rats

**Figure 1.**
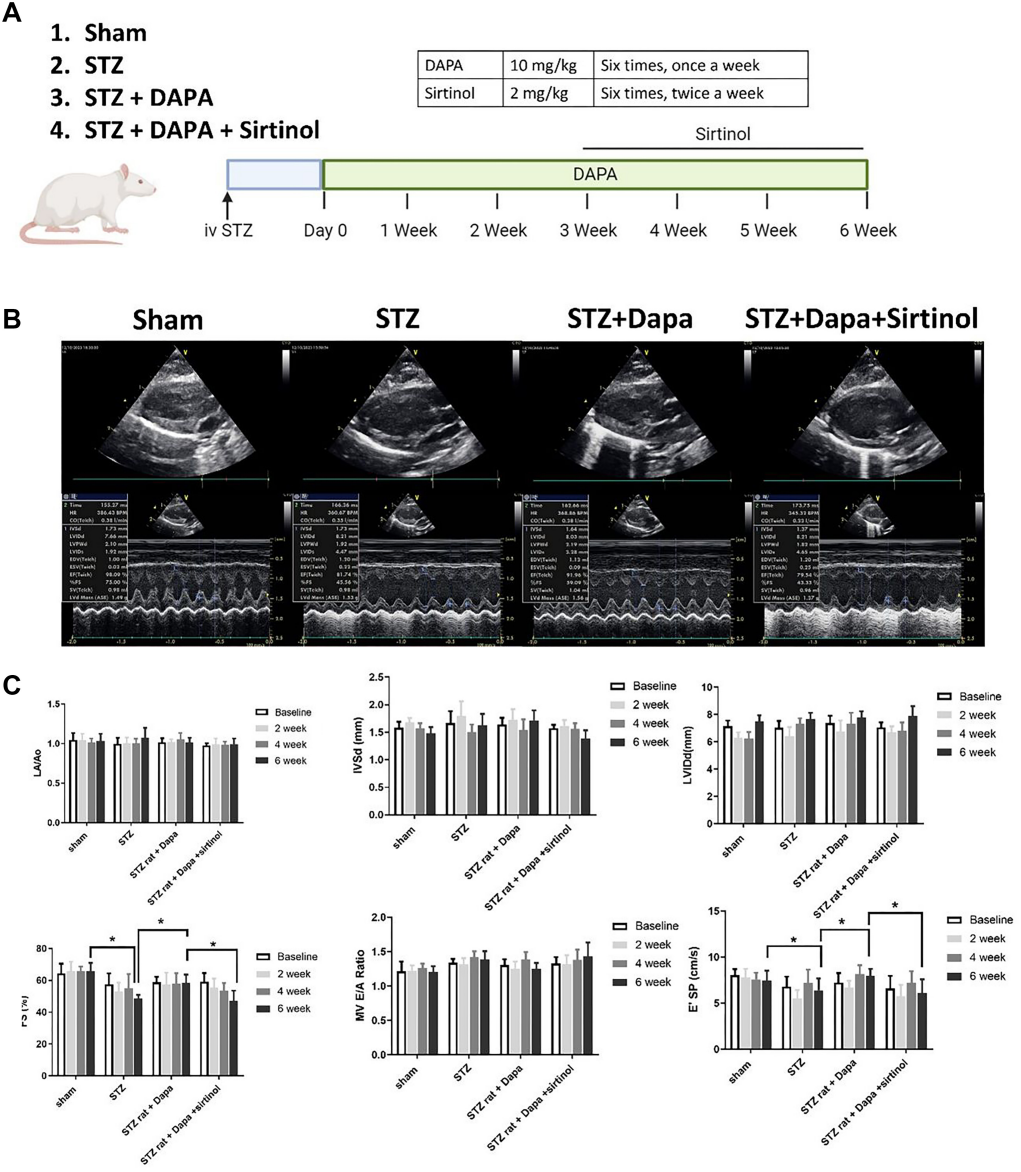
**The changes in various echocardiographic parameters over time in Sham, STZ, STZ+DAPA, and STZ+DAPA+Sirtinol treated rats.** (A) The illustration of study design; (B) The representative echocardiography images of each group; (C) Bar graphs representing measurements taken at baseline, two weeks, four weeks, and six weeks regarding left atrial diameter (LA Diam)/aortic diameter (AO Diam), IVSd, left ventricular internal diameter in diastole (LVIDd), FS, MV E/A ratio and diastolic funciton (E′). *P < 0.05 and ***P < 0.001. STZ: Streptozotocin; DAPA: Dapagliflozin; AO: Aorta; LA: Left atrial; FS: Fractional shortening; IVSd: Interventricular septal wall thickness in diastole; LVIDd: Left ventricular internal diameter in diastole; MV A Vel: Mitral valve late diastolic velocity; E’ SP: E-wave peak velocity.

**Figure S1.**
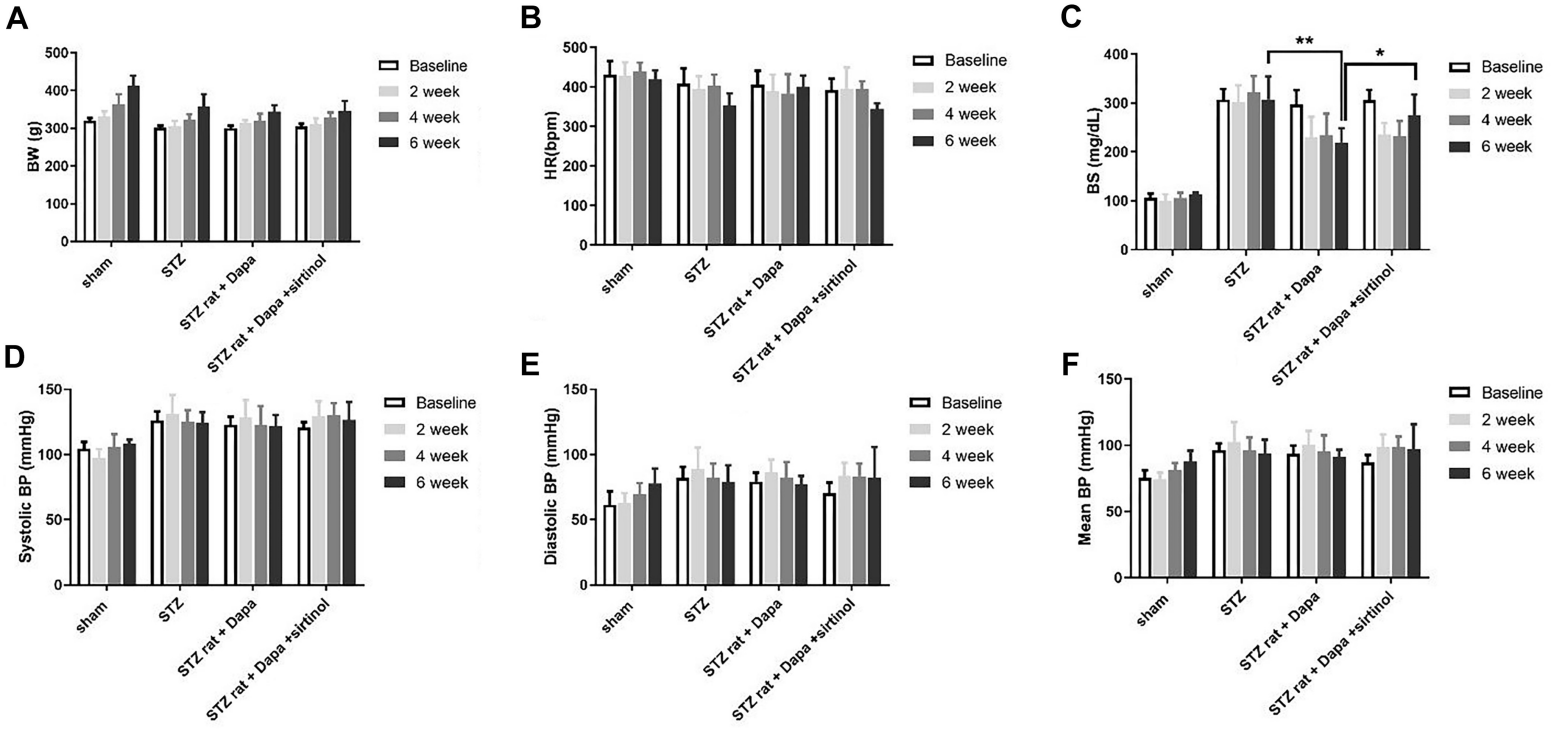
**Physiological parameters in Sham, STZ, STZ+DAPA, and STZ+DAPA+Sirtinol treated rats over time, measured at baseline, 2 weeks, 4 weeks, and 6 weeks.** (A) BW: No significant difference; (B) HR: No significant difference; (C) BS: The STZ group shows a significant increase in BS levels compared to the Sham group, indicating hyperglycemia. DAPA treatment significantly reduces BS levels, and the addition of Sirtinol slightly increases BS levels compared to DAPA treatment alone; (D) Systolic BP: No significant difference; (E) Diastolic BP: No significant difference; (F) Mean BP: No significant difference. *P < 0.05, **P < 0.01, and ***P < 0.001. STZ: Streptozotocin; DAPA: Dapagliflozin; BW: Body weight; BS: Blood sugar; HR: Heart rate; BP: Blood pressure.

**Figure S2.**
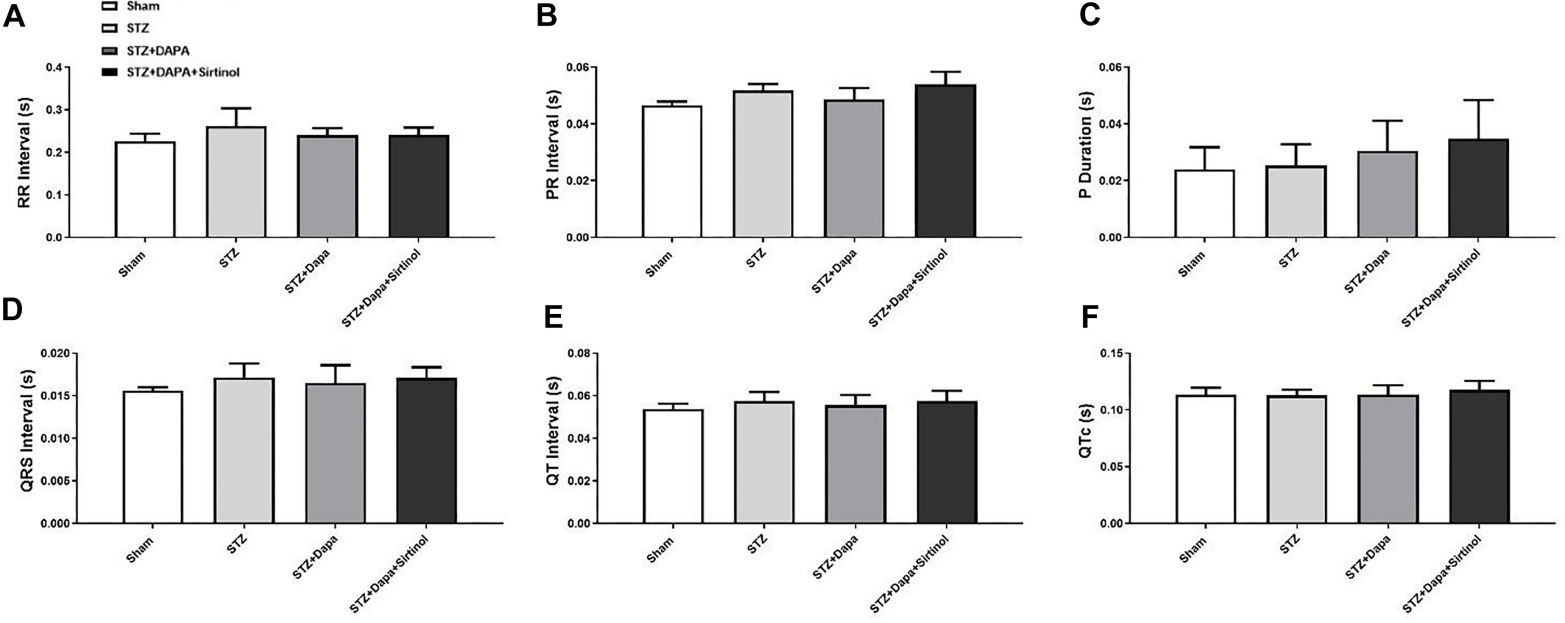
**ECG intervals in Sham, STZ, STZ+DAPA, and STZ+DAPA+Sirtinol treated rats.** (A–F) Bar graphs represent ECG intervals measured in seconds (s). The RR interval, PR interval, P duration, QRS interval, QT interval, and corrected QT interval (QTc) showed no statistically significant differences between the groups. STZ: Streptozotocin; DAPA: Dapagliflozin; ECG: Electrocardiographic.

We established the STZ-induced diabetes rat model and observed the outcomes following DAPA and Sirtinol treatment (Figure 1A). The echocardiographic parameters measured at baseline, two weeks, four weeks, and six weeks are shown in Figure 1B and 1C. The STZ group exhibited a significant decrease in FS compared to the Sham group (Sham vs STZ; *P* < 0.05), indicating impaired cardiac function. DAPA treatment (STZ+DAPA) improved FS (STZ vs STZ+DAPA; *P* < 0.05), while the addition of Sirtinol (STZ+DAPA+Sirtinol) reduced FS (STZ+DAPA vs STZ+DAPA+Sirtinol; *P* < 0.05). These results suggest that DAPA exerts a protective effect on cardiac function, which is diminished by the addition of Sirtinol, highlighting the role of SIRT1 in mediating the cardioprotective effects of DAPA under hyperglycemic conditions. The STZ group also showed a significant increase in blood glucose levels compared to the Sham group, confirming hyperglycemia (Figure S1). DAPA treatment significantly reduced blood glucose levels (STZ vs STZ+DAPA; *P* < 0.01), while the addition of Sirtinol slightly increased blood glucose levels compared to DAPA treatment alone (STZ+DAPA vs STZ+DAPA+Sirtinol; *P* < 0.05). Physiological parameters, including HR and blood pressure, showed no significant differences between the groups. Additionally, ECG intervals did not differ statistically across the groups (Figure S2).

### Hemodynamic assessment of cardiac function in Sham, STZ, STZ+DAPA, and STZ+DAPA+Sirtinol treated rats by pressure-volume loop analysis

**Figure 2.**
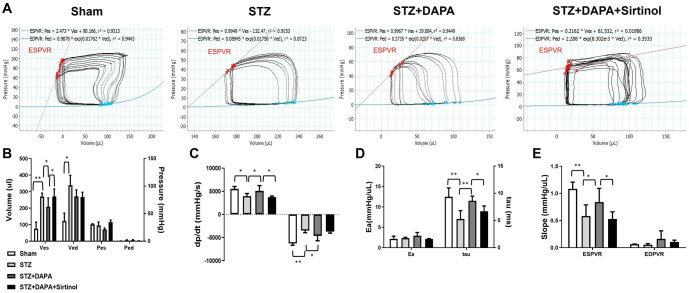
**Hemodynamic assessment of cardiac function in Sham, STZ, STZ+DAPA, and STZ+DAPA+Sirtinol treated rats by pressure–volume (PV) loop analysis**. (A) Representative PV loops for four experimental groups (Sham, STZ, STZ+DAPA, and STZ+DAPA+Sirtinol). The end-systolic PV relationship (ESPVR) and end-diastolic PV relationship (EDPVR) are shown with their respective equations and r-squared values. ESPVR and EDPVR curves indicate cardiac contractility and compliance, respectively. DAPA reversed the effect of HG, and sirtinol reversed this effect. (B) Bar graph representing volumes at end systole (Ves), end diastole (Ved), end systolic pressure (Pes), and end diastolic pressure (Ped). The results indicated a significant increase in Ves and Ved in the STZ group compared with those in the sham group, suggesting impaired cardiac function. DAPA treatment (STZ + DAPA) reduced these volumes, and the addition of sirtinol (STZ + DAPA + Sirtinol) partially reversed this effect. (C) Bar graph showing the rate of pressure change in the left ventricle during contraction (dp/dt max) and relaxation (dp/dt min). The STZ group showed significant impairments in both dp/dt max and dp/dt min compared to the sham group. DAPA treatment improved these parameters, whereas addition of sirtinol reduced them. (D and E) Bar graphs representing arterial elastance (Ea), time constant of relaxation (tau), and slopes of ESPVR and EDPVR. The STZ group showed a significant increase in tau levels, indicating impaired relaxation, which was improved by DAPA treatment. However, the addition of sirtinol diminished this improvement. The ESPVR slope was reduced in the STZ group, indicating reduced contractility, which was improved by DAPA treatment and partially reversed by sirtinol treatment. The EDPVR slope showed no significant differences among the groups. *P < 0.05 and ***P < 0.001. STZ: Streptozotocin; DAPA: Dapagliflozin; ESPVR: End-systolic pressure–volume relationship; EDPVR: End-diastolic pressure–volume relationship; Ves: End-systole; Ved: End-diastole; Pes: End-systolic pressure; Ped: End-diastolic pressure; Ea: Arterial elastance; Tau: Time constant of relaxation.

The pressure–volume (PV) loops for the four experimental groups (sham, STZ, STZ+DAPA, and STZ+DAPA+Sirtinol) are shown in Figure 2A. A significant increase in end-systolic volume (Ves) and end-diastolic volume (Ved) was observed in the STZ group compared to the sham group (Sham vs STZ; *P* < 0.01) (Figure 2B). However, DAPA treatment significantly decreased Ves levels (STZ vs STZ+DAPA; *P* < 0.05), and this effect was reversed by sirtinol treatment (STZ+DAPA vs STZ+DAPA+Sirtinol; *P* < 0.05). Similar trends were observed in the left ventricle during contraction (dp/dt max), the time constant of relaxation (tau), and the ESPVR slopes (Figure 2C–2E). HG conditions altered the PV loop parameters, increasing the volumes and reducing the pressure change rates. DAPA treatment improved these hemodynamic parameters and exerted a protective effect on cardiac function. The addition of sirtinol diminished these beneficial effects, highlighting the role of SIRT1 in mediating the cardioprotective effects of DAPA under HG conditions.

### Assessment of apoptosis and fibrosis in response to STZ-induced diabetes and the effect of DAPA and sirtinol treatments

**Figure 3.**
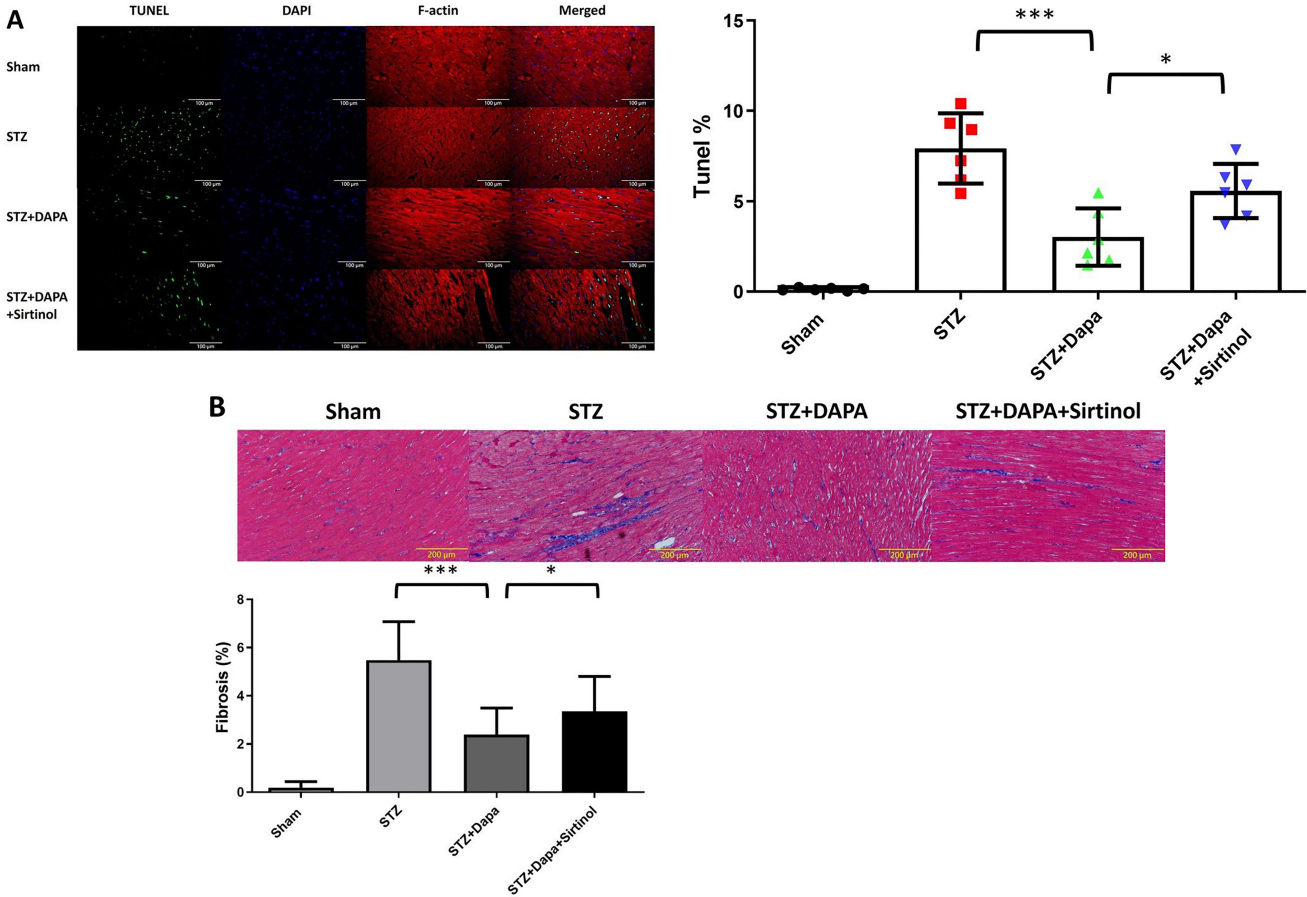
**Effect of different treatments on cellular apoptosis, histological analysis ofcardiac fibrosis**, **and inducibility and duration of AF in Sham,STZ, STZ+DAPA, and STZ+DAPA+Sirtinol treated rats.** (A) Representative immunofluorescence images showing TUNEL (green), DAPI (blue), and F-actin (red) staining in cardiac tissues under different conditions: Sham, STZ, STZ+DAPA, and STZ+DAPA+Sirtinol. The images showed increased apoptosis (TUNEL-positive cells) in the STZ group compared to that in the sham group, with a reduction observed in the STZ+DAPA and STZ+DAPA+Sirtinol groups. Quantitative analysis of TUNEL-positive cells as a percentage of the total cells. The bar graph shows a significant increase in apoptosis in the STZ group, which was markedly reduced by DAPA treatment and further attenuated by sirtinol treatment. Data are shown as mean ± SEM. (B) Representative images of Masson’s trichrome staining showing collagen deposition (blue) and muscle fibers (red) in cardiac tissue sections from the Sham, STZ, STZ+DAPA, and STZ+DAPA+Sirtinol-treated groups. The images indicated increased collagen deposition in the STZ group compared to the sham group, suggesting increased fibrosis. The DAPA treatment group showed reduced collagen deposition compared to the STZ group, indicating a protective effect of DAPA against fibrosis. The addition of Sirtinol to the HG+DAPA treatment resulted in increased collagen deposition compared with the HG+DAPA treatment alone, suggesting that sirtinol reduces the antifibrotic effect of DAPA. Quantification of the fibrotic area percentage in the different treatment groups. The results showed a significant increase in the percentage of fibrotic area in the STZ group compared to that in the sham group, indicating increased fibrosis. Treatment with DAPA significantly reduced the percentage of fibrotic area, suggesting an antifibrotic effect of DAPA. The addition of sirtinol to the HG+DAPA treatment increased the percentage of fibrotic area compared to HG+DAPA alone, indicating that sirtinol diminished the antifibrotic effect of DAPA. Data are shown as mean ± SEM. *P < 0.05, **P < 0.01, ***P < 0.001. STZ: Streptozotocin; DAPA: Dapagliflozin; AF: Atrial fibrillation; DAPI: 4’,6-Diamidino-2-phenylindole; SEM: Standard error of the mean.

Immunofluorescence images revealed a pronounced increase in TUNEL-positive apoptotic cells in the STZ group, which was significantly reduced by DAPA treatment and further diminished when sirtinol was added (STZ vs STZ+DAPA; *P* < 0.001; STZ+DAPA vs STZ+DAPA+Sirtinol; *P* < 0.05) (Figure 3A). Representative images of Masson’s trichrome staining showed increased collagen deposition in the STZ group compared to the sham group, suggesting elevated fibrosis (Figure 3B). DAPA treatment reduced collagen deposition relative to the STZ group, indicating a protective effect against fibrosis. The addition of sirtinol, however, resulted in increased collagen deposition compared to the STZ+DAPA group, suggesting that sirtinol attenuates the antifibrotic effect of DAPA (STZ vs STZ+DAPA; *P* < 0.001; STZ+DAPA vs STZ+DAPA+Sirtinol; *P* < 0.05). These findings suggest that DAPA may mitigate cardiac fibrosis by modulating SIRT1 activity.

### SIRT1 mediated the anti-arrhythmic effects of DAPA under HG conditions

**Figure 4.**
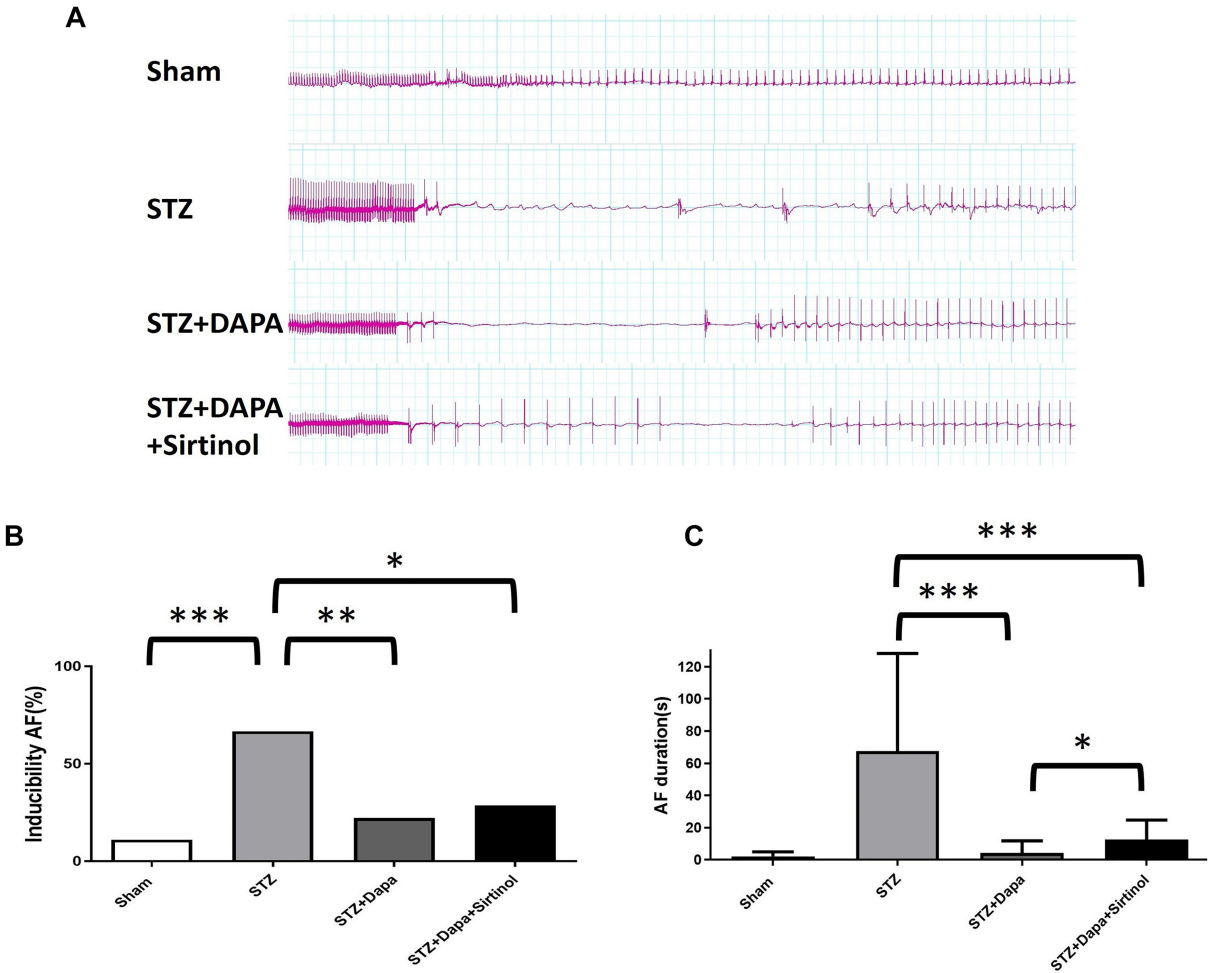
**Impact of DAPA and Sirtinol on atrial fibrillation inducibility and duration in STZ-induced diabetic rats.** (A) Representative ECG tracings showing AF episodes in sham, STZ, STZ+DAPA, and STZ+DAPA+Sirtinol groups. The tracings indicated more frequent and longer AF episodes in the STZ group than in the sham group. Treatment with HG + DAPA reduced the frequency and duration of AF episodes. The addition of sirtinol to the HG+DAPA treatment resulted in increased AF episodes compared to HG+DAPA alone. (B) A bar graph representing the percentage of AF inducibility in the different treatment groups. The results showed a significant increase in AF inducibility in the STZ group compared to that in the sham group, indicating a higher susceptibility to AF. Treatment with HG + DAPA significantly reduced AF inducibility, suggesting a protective effect of DAPA. The addition of sirtinol to the HG+DAPA treatment increased AF inducibility compared to HG+DAPA treatment alone, indicating that sirtinol diminishes the protective effect of DAPA. Data are shown as mean ± SEM. (C) A bar graph representing the duration of AF episodes in seconds for each group. Data are shown as mean ± SEM. The results showed a significant increase in the duration of AF episodes in the STZ group compared to that in the sham group, indicating prolonged AF. Treatment with HG + DAPA significantly reduced the duration of AF episodes, suggesting a beneficial effect of DAPA. The addition of sirtinol to the HG+DAPA treatment increased the duration of AF episodes compared with HG+DAPA treatment alone, indicating that sirtinol reduces the beneficial effect of DAPA. Data are shown as mean ± SEM. *P < 0.05, **P < 0.01, ***P < 0.001. STZ: Streptozotocin; DAPA: Dapagliflozin; AF: Atrial fibrillation.

The STZ group exhibited more frequent and longer AF episodes compared to the sham group (Figure 4A). Treatment with DAPA reduced both the frequency and duration of AF episodes, while the addition of sirtinol increased the number of AF episodes compared to the STZ+DAPA group. A significant increase in AF inducibility was observed in the STZ group relative to the sham group, indicating a higher susceptibility to AF (Sham vs STZ; *P* < 0.001; STZ vs STZ+DAPA; *P* < 0.01; STZ vs STZ+DAPA+Sirtinol; *P* < 0.05) (Figure 4B). Similarly, the STZ group showed a significant increase in the duration of AF episodes compared to the sham group, suggesting prolonged AF (STZ vs STZ+DAPA; *P* < 0.001; STZ vs STZ+DAPA+Sirtinol; *P* < 0.001; STZ+DAPA vs STZ+DAPA+Sirtinol; *P* < 0.05) (Figure 4C). DAPA treatment significantly reduced both the inducibility and duration of AF episodes, suggesting a beneficial effect. However, the addition of sirtinol counteracted this effect by increasing the duration of AF episodes, indicating that sirtinol diminishes the protective effects of DAPA. This highlights the role of SIRT1 in mediating the antiarrhythmic effects of DAPA under hyperglycemic conditions.

### Effects of dapagliflozin and HG on cell viability and SIRT1 expression

**Figure 5.**
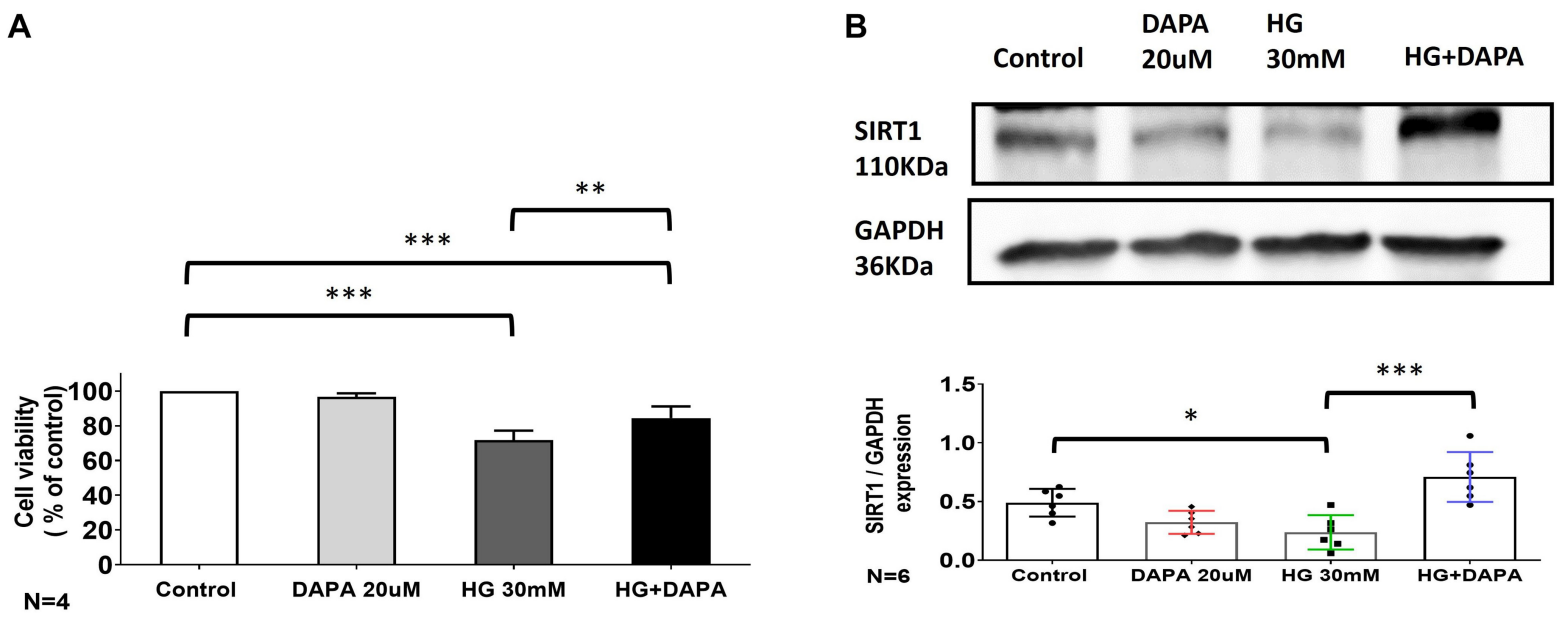
**Effects of Dapagliflozin (DAPA) and HG on cell viability and SIRT1 expression.** (A) Cell viability was assessed by 2,5-diphenyl-2H-tetrazolium bromide (MTT) assay. The bar graph represents cell viability (as a percentage of the control) after treatment with DAPA (20 µM), HG (30 mM), or HG combined with DAPA. This significant decrease in HG levels was reversed by DAPA treatment. Data are shown as mean ± SEM, *N* ═ 4; (B) Representative western blot images showing SIRT1 (110 kDa) and GAPDH (36 kDa) protein levels in the control, DAPA-, HG-, and HG+DAPA-treated cells. SIRT1 protein expression was quantified and normalized to that of GAPDH. Increased SIRT1 expression was observed in the HG group and was reversed by DAPA treatment. Data are presented as mean ± SEM, *N* ═ 6. *P < 0.05, **P < 0.01, ***P < 0.001. MTT: 2,5-diphenyl-2H-tetrazolium bromide; SIRT1: Sirtuin 1; DAPA: Dapagliflozin; HG: High glucose; SEM: Standard error of the mean.

To further investigate the effect of SIRT1 on HG-induced cardiac dysfunction, apoptosis, and AF, we assessed cell viability in HL1 cells treated with DAPA (20 µM), HG (30 mM), and a combination of HG and DAPA using the MTT assay (Figure 5A). Cell viability was significantly reduced under HG conditions but was improved after DAPA treatment (Control vs HG; *P* < 0.001; Control vs HG+DAPA; *P* < 0.001; HG vs HG+DAPA; *P* < 0.01). The levels of SIRT1 protein in the different treatment groups showed a significant decrease in the HG group compared to the control and HG+DAPA groups (Control vs HG; *P* < 0.05; HG vs HG+DAPA; *P* < 0.001) (Figures 4B and 4C and 5B). These results suggest that DAPA enhances SIRT1 expression under HG conditions.

### Effects of dapagliflozin, HG, and sirtinol on SIRT1 and the expression apoptosis markers

**Figure 6.**
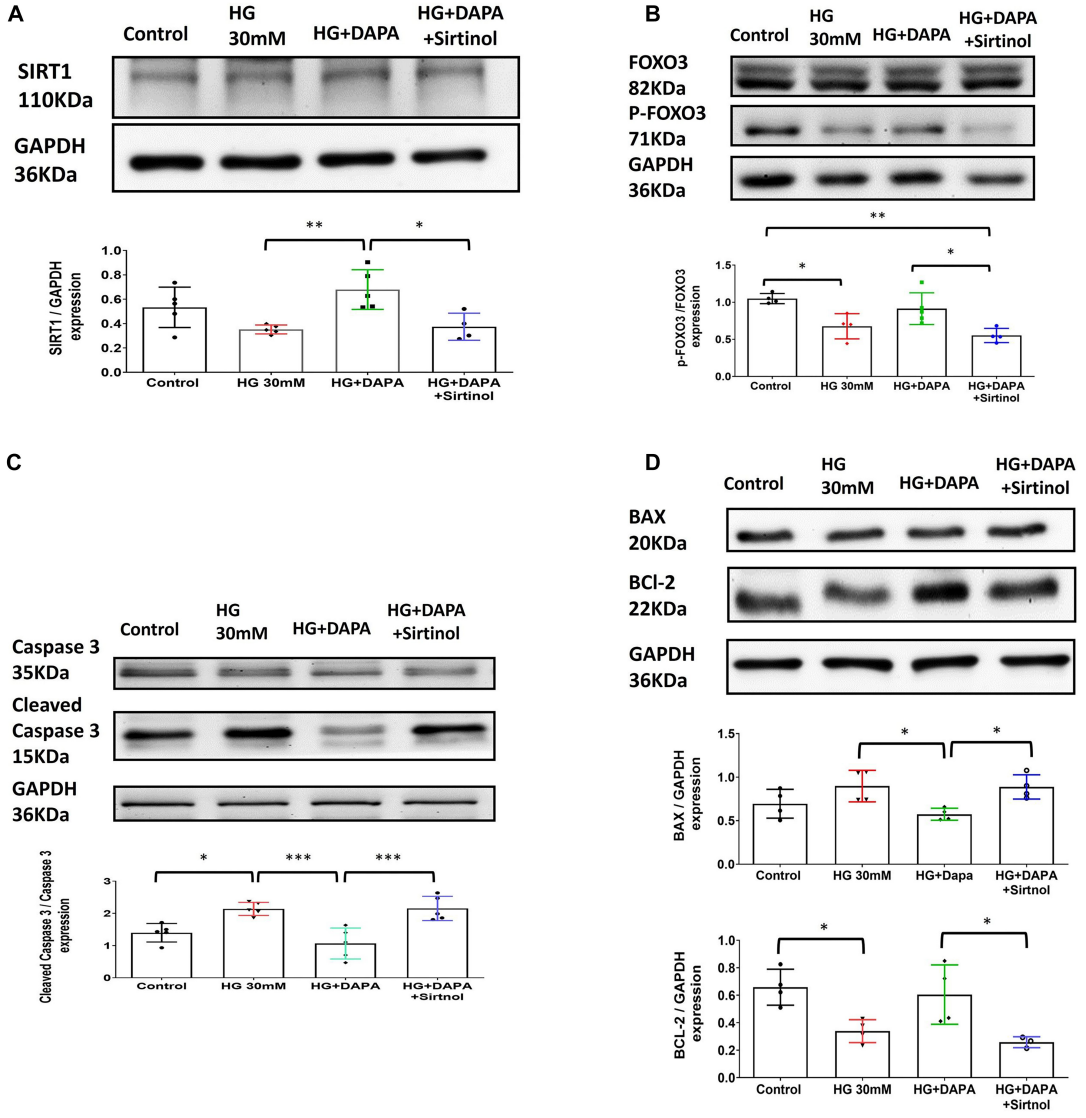
**Effects of Dapagliflozin (DAPA), HG, and sirtinol on SIRT1, FOXO3, Cleaved Caspase 3, and BAX/BCL-2 expression and phosphorylation.** (A) Western blot analysis of SIRT1 (110 kDa) and GAPDH (36 kDa) protein levels in control, HG (30 mM)-, HG+DAPA-, and HG+DAPA+Sirtinol-treated cells. SIRT1 protein expression was reversed using small interfering RNA (siRNA) targeted against SIRT1 mRNA (SIRT1 inhibitors). Data are shown as mean ± SEM. (B) Western blot analysis of FOXO3 (82 kDa), phosphorylated FOXO3 (P-FOXO3, 71 kDa), and GAPDH (36 kDa) protein levels in control, HG (30 mM)-, HG+DAPA-, and HG+DAPA+Sirtinol-treated cells. The ratio of P-FOXO3 to total FOXO3 protein levels was significantly decreased in the HG group compared to the control and in the HG+DAPA group compared to the HG+DAPA+Sirtinol group. Data are shown as mean ± SEM. (C) Western blot analysis showing Caspase 3 (35 kDa), Cleaved Caspase 3 (15 kDa), and GAPDH (36 kDa) protein levels in control, HG (30 mM)-, HG+DAPA-, and HG+DAPA+Sirtinol-treated cells. The ratio of Cleaved Caspase 3 to total Caspase 3 protein levels significantly increased in the HG group compared to the control group, which was reversed by DAPA treatment and then reversed again by sirtinol treatment. Data are presented as mean ± SEM. (D) Western blot analysis showing BAX (20 kDa), BCL-2 (22 kDa), and GAPDH (36 kDa) protein levels in control, HG (30 mM)-, HG+DAPA-, and HG+DAPA+Sirtinol-treated cells. BAX protein expression significantly decreased after DAPA treatment and was significantly reversed after sirtinol treatment. BCL-2 protein expression was significantly decreased in the HG group compared to that in the Sham group and in the STZ+DAPA group compared to that in the STZ+DAPA+Sirtinol group. Data are shown as mean ± SEM. *P < 0.05, **P < 0.01, ***P < 0.001. SIRT1: Sirtuin 1; DAPA: Dapagliflozin; HG: High glucose; GADPH: Glyceraldehyde-3-phosphate dehydrogenase; FOXO3: Forkhead box O3; SEM: Standard error of the mean.

Based on the results from the DAPA and Sirtinol treatments in the STZ-induced diabetic rat model, we established an HL-1 cell model under high-glucose (HG) conditions to investigate the effects of SIRT1. The addition of Sirtinol to the HG+DAPA treatment reduced SIRT1 expression, confirming its inhibitory effect on SIRT1 (HG vs HG+DAPA; *P* < 0.01; HG+DAPA vs HG+DAPA+Sirtinol; *P* < 0.05) (Figure 6A). In the HG group, FOXO3 phosphorylation decreased significantly, indicating suppressed FOXO3 activity (Control vs HG; *P* < 0.05; Control vs HG+DAPA+Sirtinol; *P* < 0.01; HG+DAPA vs HG+DAPA+Sirtinol; *P* < 0.05) (Figure 6B). DAPA treatment restored FOXO3 activity, but Sirtinol reversed this effect. Similarly, cleaved Caspase 3 levels were elevated in the HG group, suggesting increased apoptosis, while DAPA reduced these levels, demonstrating its anti-apoptotic effect. However, Sirtinol counteracted this effect, increasing cleaved Caspase 3 levels again (Control vs HG; *P* < 0.05; HG vs HG+DAPA; *P* < 0.001; HG+DAPA vs HG+DAPA+Sirtinol; *P* < 0.001) (Figure 6C). The HG group also exhibited a pro-apoptotic shift, with increased BAX and decreased BCL-2 expression. DAPA reversed this shift, but Sirtinol partially reversed DAPA’s protective effects (BAX: HG vs HG+DAPA; *P* < 0.05; HG+DAPA vs HG+DAPA+Sirtinol; *P* < 0.05; BCL-2: Control vs HG; *P* < 0.05; HG+DAPA vs HG+DAPA+Sirtinol; *P* < 0.05) (Figure 6D). Overall, Sirtinol reduced the protective effects of DAPA, highlighting the role of SIRT1 in regulating apoptosis under HG conditions.

### Effects of dapagliflozin, HG, and sirtinol on autophagy and mitophagy markers in treated cells

**Figure 7.**
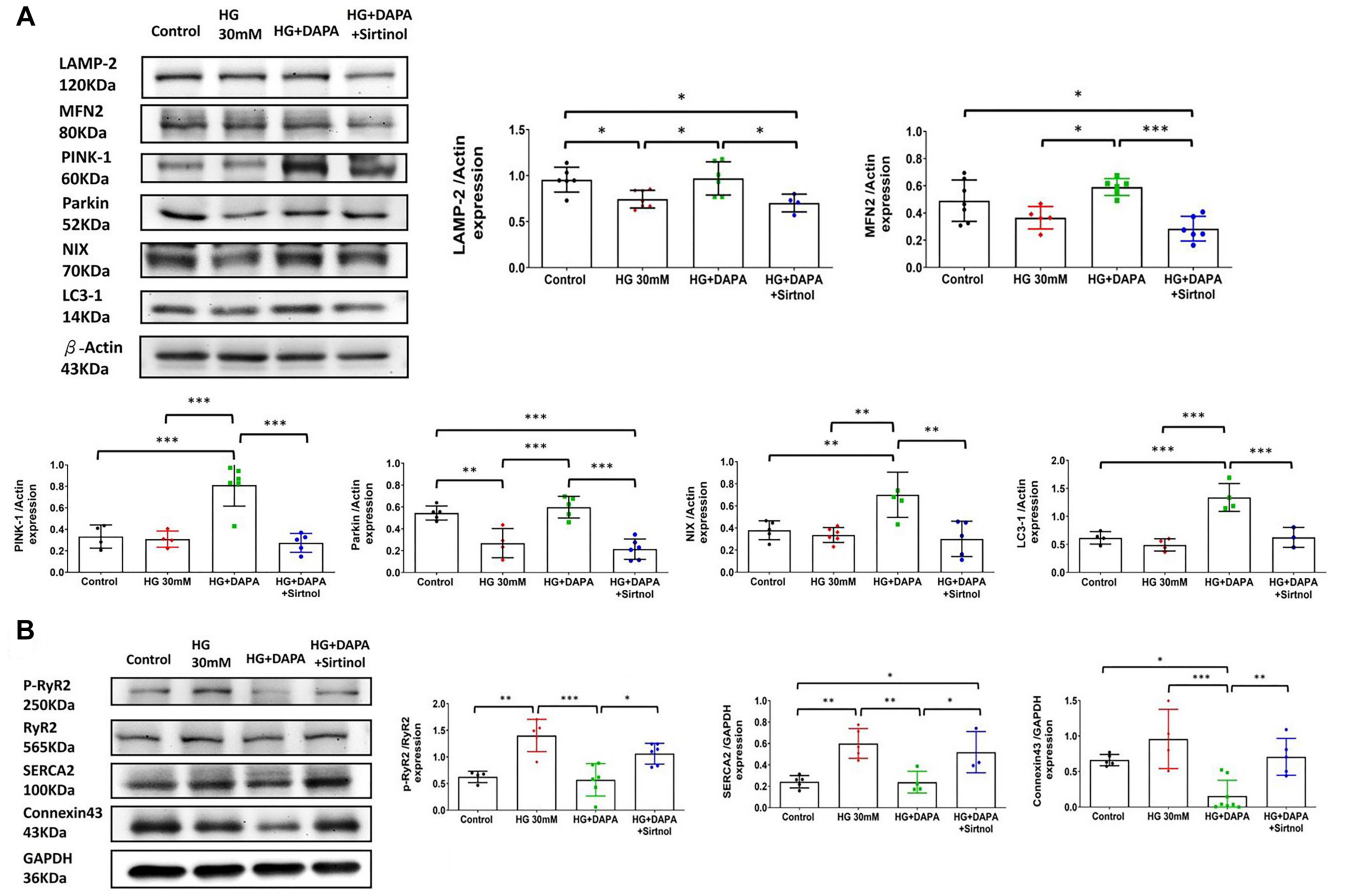
**Effects of Dapagliflozin (DAPA), HG, and sirtinolon autophagy, mitophagy markers, and cardiac calcium handling proteins in treated cells.** (A) Western blot analysis of LAMP-2 (120 kDa), MFN2 (80 kDa), PINK-1 (60 kDa), Parkin (52 kDa), NIX (70 kDa), LC3-1 (14 kDa), and β-Actin (43 kDa) protein levels in control, HG (30 mM), HG+DAPA, and HG+DAPA+Sirtinol treated cells. Bar graphs represent the quantification of protein expression for LAMP-2, MFN2, PINK-1, Parkin, NIX, and LC3-1 normalized to β-Actin. There was a significant difference in the LAMP-2 and Parkin levels between the HG (30 mM) and control groups. After DAPA treatment, there were significant differences in LAMP-2, MFN2, PINK-1, Parkin, NIX, and LC3-1 protein levels in the HG+DAPA group compared to those in the HG (30 mM) group. After sirtinol treatment, a significant reverse phenomenon was noted for LAMP-2, MFN2, PINK-1, Parkin, NIX, and LC3-1 when the HG+DAPA+Sirtinol group was compared to the HG+DAPA group. Data are shown as mean ± SEM. (B) Western blot analysis of phosphorylated RyR2 (P-RyR2, 250 kDa), RyR2 (565 kDa), SERCA2 (100 kDa), Connexin43 (43 kDa), and GAPDH (36 kDa) protein levels in control, HG (30 mM)-, HG + DAPA-, and HG + DAPA + sirtinol-treated cells. Bar graphs represent the quantification of protein expression for the P-RyR2/RyR2 ratio, SERCA2, and Connexin43 normalized to GAPDH. There was a significant difference in the P-RyR2/RyR2 ratio and SERCA2 expression compared to the control group. After DAPA treatment, there was a significant difference in the P-RyR2/RyR2 ratio, SERCA2, and Connexin43 protein expression levels between the HG+DAPA and HG (30 mM) groups. After sirtinol treatment, a significant reverse phenomenon was noted in the P-RyR2/RyR2 ratio, SERCA2, and Connexin43 expression levels when the HG+DAPA+Sirtinol group was compared to the HG+DAPA group. Data are shown as mean ± SEM. *P < 0.05, **P < 0.01, ***P < 0.001. SIRT1: Sirtuin 1; DAPA: Dapagliflozin; HG: High glucose; LAMP-2: Lysosome-associated membrane protein 2; MFN2: Mitofusin-2; PINK-1: PTEN-induced kinase-1; LC3-1: Light chain 3-1; RyR2: Ryanodine receptor 2; SERCA2: Sarco/endoplasmic reticulum Ca^2+^-ATPase; SEM: Standard error of mean.

SIRT1 is a crucial enzyme for both autophagy and mitophagy. In the HG group, the expression of LAMP-2 and Parkin was significantly lower compared to the control, indicating impaired autophagy and mitophagy (Figure 7A). DAPA treatment restored the expression of LAMP-2, PINK-1, NIX, MFN2, Parkin, and LC3-I, suggesting a protective role in promoting these processes. However, Sirtinol reversed these beneficial effects, as demonstrated by the comparison between the HG + DAPA + Sirtinol and HG + DAPA groups, underscoring the key role of SIRT1 in regulating autophagy and mitophagy under HG conditions.

### SIRT1 impacted cardiac calcium handling proteins in cells exposed to HG conditions

A deficiency in SIRT1 may impair the regulation of intracellular Ca^2+^ in cardiomyocytes, potentially leading to arrhythmias [22]. In our HG cell model, we observed a significant increase in the P-RyR2/RyR2 ratio and connexin43 levels in the HG group (Figure 7B). DAPA treatment reduced the P-RyR2/RyR2 ratio, SERCA2, and connexin43, while Sirtinol reversed these effects by increasing their levels. HG conditions impaired the expression and phosphorylation of key calcium-handling proteins, including RyR2, SERCA2, and connexin43, which could disrupt calcium regulation in cardiac cells. DAPA helped normalize these proteins, but Sirtinol diminished its protective effects, highlighting the key role of SIRT1 in regulating calcium handling under HG conditions.

### Evaluation of cellular apoptosis and reactive oxygen species levels under HG conditions and the effects of DAPA and sirtinol treatments

**Figure 8.**
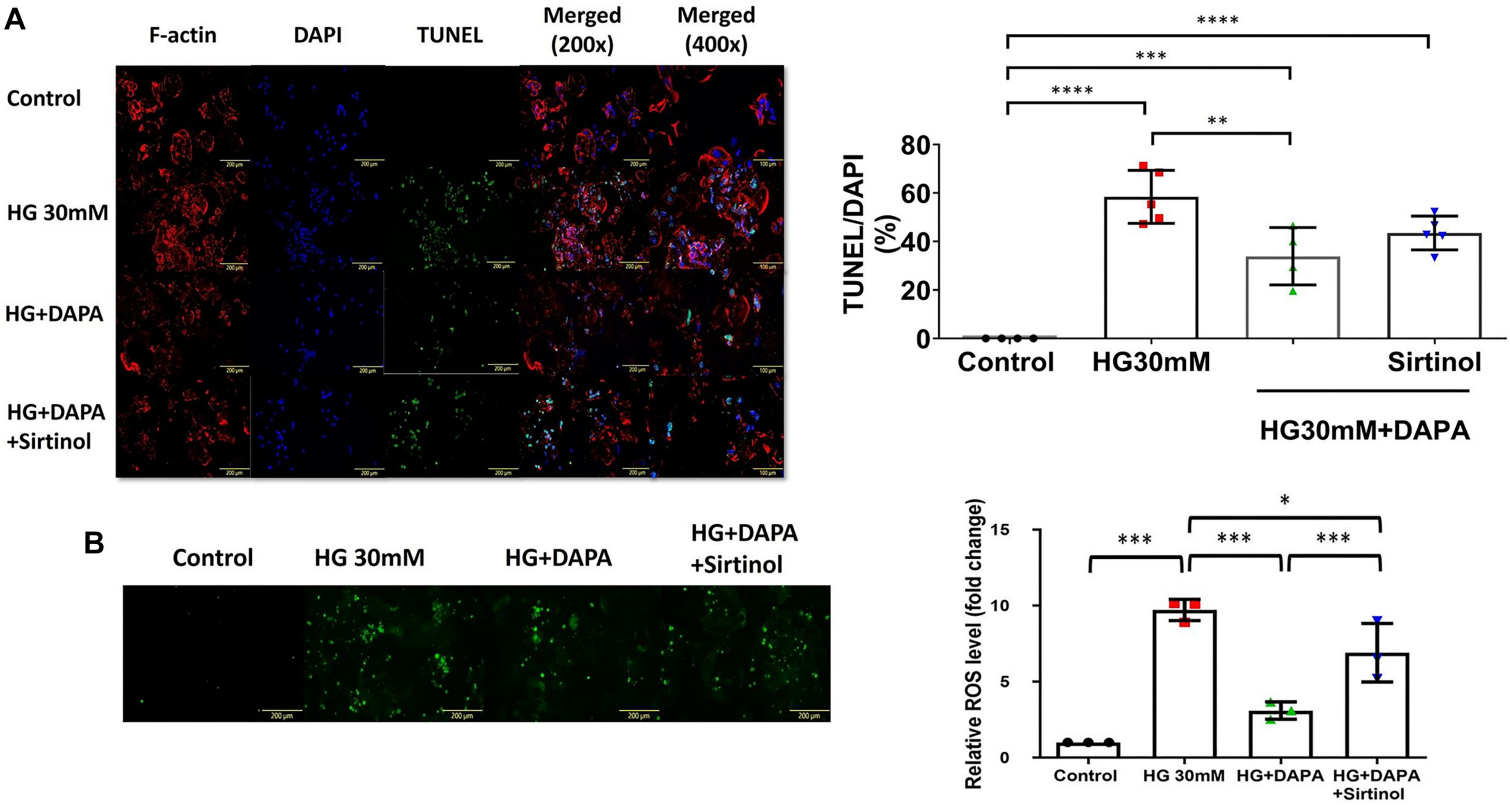
**Effect of different treatments on cellular apoptosis and ROS levels under HG conditions and the effects of DAPA and sirtinol treatments.** (A) Representative immunofluorescence images showing F-actin (red), DAPI (blue), and TUNEL (green) staining of cells under various conditions: Control, HG 30 mM, HG+DAPA, and HG+DAPA+Sirtinol. Merged images are presented at 200× and 400× magnifications, indicating the extent of apoptosis (TUNEL-positive cells) and cellular morphology. A higher percentage of apoptosis was observed under HG conditions, which decreased after DAPA treatment but was reversed by sirtinol treatment. Quantification of apoptotic cells is represented as the percentage of TUNEL-positive cells (green) normalized to DAPI (blue) staining. A significant increase in apoptosis was observed in the HG group compared to the control group. This increase was attenuated upon treatment with DAPA, but this attenuation was reversed after the addition of sirtinol. Data are shown as mean ± SEM. (B) Representative fluorescence images of ROS (green) in cells treated with control, HG 30 mM, HG+DAPA, or HG+DAPA+Sirtinol. The images indicate a substantial increase in ROS levels in the HG 30 mM group compared to the control group, which was attenuated by DAPA and further influenced by sirtinol. Quantification of ROS levels, expressed as fold change relative to the control group. The bar graph shows a significant elevation in ROS levels in the HG 30-mM group, which was significantly reduced by DAPA treatment. The addition of sirtinol to DAPA significantly increased ROS levels compared with treatment with DAPA alone. Data are shown as mean ± SEM. *P < 0.05, **P < 0.01, ***P < 0.001. DAPA: Dapagliflozin; HG: High glucose; SEM: Standard error of the mean; ROS: Reactive oxygen species.

Immunofluorescence images showed a significant increase in TUNEL-positive cells in the HG group compared to both the control and HG+DAPA groups (Control vs HG; *P* < 0.0001; HG vs HG+DAPA; *P* <0.01) (Figure 8A). No significant increase was observed following sirtinol treatment. However, DAPA treatment led to a notable reduction in apoptosis, suggesting its potential protective effect against HG-induced apoptosis. Fluorescence imaging also revealed a marked increase in ROS levels in the HG group, which was significantly reduced by DAPA treatment (HG vs HG+DAPA; *P* < 0.001) (Figure 8B). These results underscore the potential of DAPA in mitigating ROS production under HG conditions.

## Discussion

In the STZ-induced rodent model of diabetes, DAPA improved hemodynamic parameters as well as cardiac systolic and diastolic functions under hyperglycemic conditions. However, these protective effects were reversed by sirtinol. Additionally, DAPA significantly reduced atrial fibrosis and decreased both the inducibility and duration of AF, effects which were similarly reversed after sirtinol treatment. Mechanistically, DAPA increased SIRT1 levels, which reduced apoptosis, decreased ROS, and enhanced autophagy and mitophagy. DAPA also modulated calcium-handling proteins in murine atrial HL-1 cells. After sirtinol treatment, these effects were diminished, indicating that the SIRT1 inhibitor negated the protective effects of DAPA on HG-induced cellular apoptosis and ROS. These results highlight SIRT1’s crucial role in apoptosis and suggest that DAPA’s impact on SIRT1 improves calcium-handling proteins, thus mitigating associated arrhythmias. In the STZ-induced rat diabetes model, DAPA reduced the amplitude of shortening and calcium transients in ventricular myocytes, suggesting that alterations in calcium transport mechanisms may contribute to these negative inotropic effects [31]. Another SGLT2 inhibitor, empagliflozin, has been shown to reduce endoplasmic reticulum Ca^2+^ release, extracellular Ca^2+^ entry, and profibrotic activity in atrial fibroblasts [32]. SGLT2 inhibitors also decrease cytoplasmic calcium via the electrogenic sodium/calcium exchanger and influence delayed afterdepolarizations, contributing to arrhythmogenesis [33]. Reverse-mode activity of the sodium/calcium exchanger leads to calcium loading in cardiomyocytes. In this study, DAPA increased SIRT1 expression and modulated calcium-handling proteins under HG conditions, resulting in a reduction in AF inducibility and duration in the STZ rat model. Moreover, the ability of SGLT2 inhibitors to control arrhythmias is linked to several cardiovascular benefits, including reduced atrial remodeling (due to lower blood pressure and LV mass), anti-inflammatory and anti-fibrotic effects, and improved mitochondrial function and energy metabolism in the heart [34]. Therefore, SGLT2 inhibitors offer both direct and indirect control of atrial arrhythmias.

Sirtuins are a family of NAD+-dependent enzymes initially recognized for their role in regulating lifespan across a variety of organisms, including yeast, *Caenorhabditis elegans, Drosophila*, and mice [35]. SIRT1, in particular, is a key molecular regulator of lifespan. It plays a crucial role in maintaining the balance between cellular growth and survival by targeting various substrates involved in essential biological processes [36]. These substrates include proteins associated with chromatin folding, metabolic regulation, and stress responses, highlighting the broad functional impact of SIRT1 within the cell [37]. As a result, SIRT1 is implicated in aging and is associated with a range of conditions, such as cancer, cardiovascular diseases, and neurodegenerative disorders [38]. Regulation of SIRT1 has also been linked to LA fibrosis. Specifically, in a mitral regurgitation model, SIRT1 regulation was observed in the LA, suggesting its potential as a therapeutic target in conditions involving LA fibrosis [39]. Furthermore, in an animal model of complete atrioventricular block, negative regulation of SIRT1 and LA fibrosis was observed in the LA myocardium [40]. This negative regulation suggests that the involvement of SIRT1 may vary across different pathological conditions affecting the LA. Our study further investigated the regulation of SIRT1, particularly through the administration of DAPA or sirtinol. We found that this regulation influenced several cellular processes, including apoptosis, autophagy, mitophagy, and ROS generation. Collectively, these processes affect the development of fibrosis in the LA. Moreover, our findings revealed that LA fibrosis significantly contributed to the inducibility and duration of AF in the STZ-induced diabetic rat model. This underscores the interconnected role of SIRT1 in modulating both cellular health and disease progression within the LA.

Sirtinol, a known inhibitor of SIRT1, interferes with its activation. SIRT1 activation leads to the deacetylation and degradation of the Notch1 intracellular domain (NICD), which in turn suppresses the Notch1 signaling pathway—a critical regulator of endothelial-to-mesenchymal transition (EndMT) and subsequent fibrosis [41]. This suggests that the therapeutic efficacy of DAPA in reducing fibrosis and AF could be compromised if combined with a SIRT1 inhibitor. However, it is important to note that the interaction between DAPA, SIRT1, and sirtinol has been primarily explored in preclinical settings. In our STZ-induced rat diabetes model, sirtinol reversed the glucose-lowering effect of DAPA, suggesting that SIRT1 inhibition may influence blood sugar levels. This effect could potentially be mediated through indirect mechanisms, such as alterations in mitochondrial function, oxidative stress, or lipid metabolism—all of which are closely tied to insulin sensitivity and glucose regulation. Clinical data on the combined use of DAPA and SIRT1 inhibitors remain unavailable. Consequently, while theoretical insights suggest that adding a SIRT1 inhibitor might reverse DAPA’s beneficial effects on fibrosis and AF, clinical studies are essential to validate these interactions and their impact on patient outcomes.

### Limitations

First, the study was conducted on a limited number of animals, focusing on the impact of SIRT1 and DAPA in the STZ-DM rat model. This limitation may affect the generalizability of the findings and suggests the need for larger studies to confirm these results. Second, the rapid HR of the animals may have influenced the accuracy of ECG measurements, potentially introducing variability into the data. Future studies could benefit from techniques aimed at stabilizing HR for more precise measurements. Third, the study used only male Sprague–Dawley rats, which precluded an assessment of potential hormonal influences on the observed effects. Including both male and female rats in future research would provide a more comprehensive understanding of how hormonal differences may impact the results.

Fourth, using AF rat models, rather than animals with induced AF, may offer a more direct approach to investigating the effects of DAPA or SIRT1 inhibitors on the mechanisms underlying AF. Future studies may need to explore this approach to enhance the relevance of the findings.

Fifth, the impact of SIRT1 should also be investigated in non-DM models to better understand its effects in populations without diabetes. Studying the role of SIRT1 in non-DM models could provide valuable insights into its broader implications, independent of diabetes-related pathways. Sixth, the levels of DAPA and sirtinol were not measured in this study, meaning that the interaction between DAPA and sirtinol could not be assessed. The focus was primarily on evaluating changes in SIRT1 levels following treatment with DAPA and sirtinol.

Despite these limitations, this study offers valuable insights into the effects of DAPA on atrial arrhythmias in the STZ-DM model. The findings highlight the potential of DAPA as a therapeutic agent for managing diabetes-associated atrial arrhythmias through the regulation of SIRT1 expression. The study suggests that SIRT1 may exert beneficial effects in controlling atrial fibrosis by regulating apoptosis, autophagy, ROS generation, and calcium channel activity. These effects could help explain the shorter duration and lower inducibility of AF observed in the study.

Together, these insights underscore the importance of further research into the role of SIRT1 in cardiac health and disease, particularly in the context of diabetic cardiomyopathy and atrial arrhythmias.

## Conclusion

SIRT1 may play a protective role in cell models by reducing apoptosis, modulating autophagy and mitophagy, and influencing calcium channel activity. In the STZ rat model, dapagliflozin decreased both the inducibility and duration of AF, potentially by upregulating SIRT1 levels. This effect may be linked to changes in calcium channel activity.

## Supplemental data

## Data Availability

Data generated or analyzed during this study are available from the corresponding author upon reasonable request.
